# Cardiac and digestive forms of chronic Chagas disease in Brazilian social welfare, 2004-2016

**DOI:** 10.47626/1679-4435-2022-1038

**Published:** 2024-02-16

**Authors:** Jean Ezequiel Limongi, Izabela Lima Perissato, Antônio Marcos Machado de Oliveira, Keile Aparecida Resende Santos

**Affiliations:** 1 Curso de Saúde Coletiva, Universidade Federal de Uberlândia, Uberlândia, MG, Brazil; 2 Instituto Nacional do Seguro Social, Uberlândia, MG, Brazil

**Keywords:** Chagas cardiomyopathy, Chagas disease, social welfare, social security, social support, cardiomiopatia chagásica, doença de Chagas, seguridade social, previdência social, apoio social

## Abstract

**Introduction:**

Chagas disease is a neglected tropical disease with a chronic clinical course and high rates of morbidity and mortality. Despite a drastic reduction in the disease’s incidence in Brazil in recent decades, older cases still impact the national social welfare system.

**Objectives:**

To analyze the sociodemographic characteristics of Brazilian social welfare beneficiaries affected by the cardiac and digestive forms of chronic Chagas disease between 2004 and 2016.

**Methods:**

This cross-sectional study was based on data from the Brazilian Ministry of Labor and Social Security. Crude and adjusted odds ratios were estimated using logistic regression.

**Results:**

Benefits were granted to 25,085 affected individuals, mostly men (15,812; 63%) with the cardiac form (20,424; 81.4%) who resided in urban areas (16,051; 64%). The highest relative frequency of benefits were granted in the Midwest macroregion (31.1/100,000 inhabitants). Male sex (odds ratios = 1.2; 95% CI 1.1-1.2), age 30-49 years (odds ratios = 1.8; 95% CI 1.4-2.1), residence in rural areas (odds ratios = 1.6; 95% CI 1.5-1.7) or the Southeast macroregion (odds ratios = 2.9; 95% CI 2.4-3.4) had the highest association with the cardiac form. Individuals with the cardiac form had a higher median age at disease onset (45 years; p < 0.001) but a lower age at work disability onset (50 years; p = 0.01).

**Conclusions:**

The impact of Chagas disease on Brazilian social welfare is mainly due to chronic Chagas cardiomyopathy, which was mainly associated with men in their productive years who live in rural areas in Southeastern Brazil.

## Abstract (pt)

**Introdução:**

A doença de Chagas é uma doença tropical negligenciada, de evolução crônica e com elevada morbimortalidade. Apesar da drástica redução na incidência da doença nas últimas décadas no Brasil, casos infectados no passado ainda impactam o sistema de seguridade social brasileiro.

**Objetivos:**

Analisar as características sociodemográficas de beneficiários da seguridade social brasileira acometidos pela doença de Chagas crônica nas formas clínicas cardíaca e digestiva no período de 2004 a 2016.

**Métodos:**

Estudo transversal com dados do Ministério do Trabalho e Previdência Social brasileiro. Empregou-se regressão logística para estimar *odds ratio* brutas e ajustadas.

**Resultados:**

Houve concessão de 25.085 benefícios, a maioria relacionada à forma cardíaca da doença de Chagas (20.424; 81,4%), ao sexo masculino (15.812; 63%) e residentes em áreas urbanas (16.051; 64%). A macrorregião Centro-Oeste apresentou maior frequência relativa de benefícios (31,1/100.000 habitantes). Sexo masculino (*odds ratio* = 1,2; IC95% 1,1-1,2), faixa etária entre 30 e 49 anos (*odds ratio* = 1,8; IC95% 1,4-2,1), residência em áreas rurais (*odds ratio* = 1,6; IC95% 1,5-1,7) ou na macrorregião Sudeste (*odds ratio* = 2,9; IC95% 2,4-3,4) foram as categorias das variáveis mais associadas à forma cardíaca. Indivíduos com a forma cardíaca apresentaram idade mediana maior no início da doença (45 anos; p < 0,001), porém menor no início da incapacidade laboral (50 anos; p = 0,01).

**Conclusões:**

O impacto da doença de Chagas na seguridade social brasileira decorre principalmente por causa da cardiomiopatia chagásica crônica. Essa forma clínica esteve associada principalmente a pessoas do sexo masculino, em idade produtiva importante, residentes em áreas rurais e da macrorregião Sudeste do Brasil.

## INTRODUCTION

Chagas disease (CD) is caused by the protozoan *Trypanosoma cruzi*, whose vector transmission occurs exclusively in rural/wild environments in Latin America.^1^ In Brazil, success combating vectors, improved sanitary conditions in housing, increased urbanization, and a rural exodus in recent decades have changed the epidemiological patern of CD. The predominantly rural profile and high incidence of the disease has been transformed into an urban situation involving older cases. However, due to the disease’s chronicity, its prevalence remains significant, especially among adults and older adults.^1,2,3^

A 2014 systematic review and meta-analysis estimated that around 4.6 million Brazilians are infected with CD.^2^ Approximately 60% of cases remain in the undetermined form, while 30% and 10% progress to the cardiac and digestive (megaesophagus and megacolon) forms, respectively.^4^ A combined cardiodigestive form is also relatively common, and the nervous system, as well as other organs, can be affected, although this is infrequent.^5^

The 1988 Brazilian Constitution guarantees social welfare as a way of ensuring rights related to health, social assistance, and social security. While health care and social assistance benefits are non-contributory, social security is contributory and mandatory.^6^

People with chronic CD frequently use health services, especially those with more complex cases. The disease also has an important psychological impact, inducing fear, stress, anxiety, low self-esteem, and depression.^5,7^ Primary care services are strongly recommended for longitudinal monitoring of non-severe, stable chronic patients, non-severe acute cases, and indeterminate cases.^5,8,9,10^

Individuals enrolled in social security who become disabled are entitled to social security benefits, the most common of which are temporary disability benefits and retirement benefits due to permanent disability, while those not enrolled in social security can receive assistance benefits if they meet the necessary requirements.^11^

Notification of chronic CD cases only became mandatory nationwide in 2020.^12^ The states of Goiás and Minas Gerais made reporting obligatory in 2013 and 2018, respectively.^13,14^ However, to date, there is no specific notification form, no official data are available, and there is no standardized flow and/or incentive for monitoring and treating cases.

The literature on CD and social welfare in Brazil is limited to old publications (before the 1980s) and small case series.^15,16,17^ The present study provides a nationwide and up-to-date history of this relationship, in addition to an approximate characterization of the national context of chronic CD, which has never been atempted. Tus, this study analyzed the sociodemographic characteristics of Brazilian social welfare beneficiaries affected by chronic CD in its cardiac and digestive clinical forms.

## METHODS

This cross-sectional study was based on secondary data from the Ministry of Labor and Social Security’s Unified Benefits Information System (SUIBE). SUIBE is not freely accessible to the public and contains socio-demographic data on beneficiaries and data related to the granting of benefits. Data access was granted afer a request to the central level of the National Social Security Institute (INSS).

The study included INSS beneficiaries who received assistance or social security benefits between 2004 and 2016, granted afer diagnosis of Chagas disease (ICD 10 B57) in the following classifications: B57.2 (Chronic form with cardiac involvement); B57.3 (Chronic form with digestive system involvement); K23.1 (Megaesophagus in Chagas disease) and K93.1 (Megacolon in Chagas disease).

The following variables were analyzed: sex, age at onset (years), length of disability (years), length of benefits (years), time elapsed between illness onset and disability (days), specific ICD code (B57.2, B57.3, K23.1, or K93.1), clinical form of CD (cardiac or digestive), age group (<29 years, 30-49 years, 50-59 years, or ≥60), area of residence (urban or rural), type of work activity (commercial, farming, or other), social security status (self-employed, special case, employed, unemployed, or other), type of benefits received (social assistance or social security), type of benefit granted (temporary disability benefits, permanent disability benefits [retirement], assistance for people with disabilities, or other), geographic macroregion (South, Southeast, Midwest, North, or Northeast), and the year benefits were granted (2004 to 2016).

Epi Info 7.2.2 (U.S. Centers for Disease Control and Prevention, Atlanta, GA, USA) was used for all statistical analyses. Logistic regression was used to determine the association between independent variables and the clinical form of the disease. Sex, age group, area of residence, geographic macroregion, and benefit type were the independent variables, while clinical form of CD was the dependent variable. This variable was defined as 1 = cardiac form or 0 = digestive form.

Two different models were used: (1) a model with individual variables, in which each independent variable was analyzed in relation to the dependent variable, ie, a sequence of bivariate analyses; and (2) the complete model including the 5 independent variables. The reference categories for each independent variable were those with the lowest frequency of cardiac form. The results of the logistic regression were presented using the crude and adjusted odds ratio (OR) and 95% CI. For continuous variables, homoscedasticity was assessed using the Bartlet test. The Kruskal-Wallis test was used for comparisons between groups of cardiac and digestive cases, and descriptive statistics were presented using medians and quartiles.

The Quantum Geographic Information System were used to spatialize the data. The frequency of benefits related to cardiac, digestive, or general (cardiodigestive) forms was adjusted for every 100,000 inhabitants for comparison among states and geographic macroregions. Brazilian Institute of Geography and Statistics (IBGE) population estimates from 2016 was used according to the following formulas:

(i) Macroregion:

Benefits (cardiac, digestive, or general form)


$$=\frac{\text{Total specific benefits granted in the microregion}}{\text{Estimated population of the macroregion}}\times 100,000\text{inhabitants}$$

(ii) State:

Benefits (cardiac, digestive, or general form)


$$=\frac{\text{Total specific benefits granted in the state}}{\text{Estimated population of the state}}\times 100,000\text{inhabitants}$$

This study was approved by the Federal University of Uberlandia Human Research Ethics Commitee (number 1,560,139/2016).

## RESULTS

A total of 25,085 people with the cardiac and digestive forms of CD received social security and/or social assistance benefits, of whom 20,424 (81.4%) had the cardiac form (ICD B57.2) and 2888 (11.5%) had the digestive form without a specific affected organ being reported (ICD B57.3). Megaesophagus (ICD K23.1) and megacolon (ICD K93.1) were specified, in 1219 (4.9%) and 554 (2.2%) cases, respectively.

The majority of beneficiaries lived in urban areas (16,051; 64%) and were men (15,812; 63%). The main work activities of the beneficiaries were commerce (14,394; 57.4%), and farming (9034; 36%). There were 10,391 (41.4%) and 10,791 (43%) beneficiaries aged 30-49 and 50-59 years, respectively.

The main benefit types were assistance due to temporary disability (15,861; 63.2%), retirement due to permanent disability (7449; 29.7%) and support for people with disabilities (1672; 6.7%). A total of 12,107 (76.3%) and 6868 (92.2%) of those with the cardiac form received benefits for temporary and permanent disability, respectively.

The most frequent forms of social security status were special cases (8837; 35.2%), employed (5569; 22.2%), unemployed (5056; 20.2%), and self-employed (4169; 16.6%).

**Figure 1:**
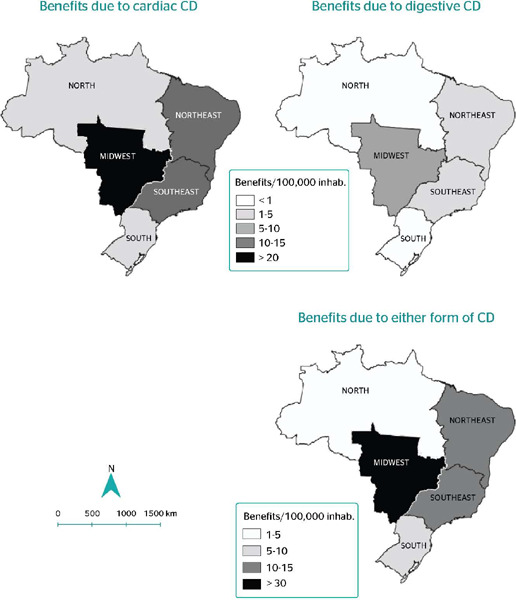
Distribution of benefits to people with Chagas disease (CD) per 100,000 inhabitants, according to geographic macroregion and clinical form of the disease, Brazil, 2004-2016.

**Figure 2:**
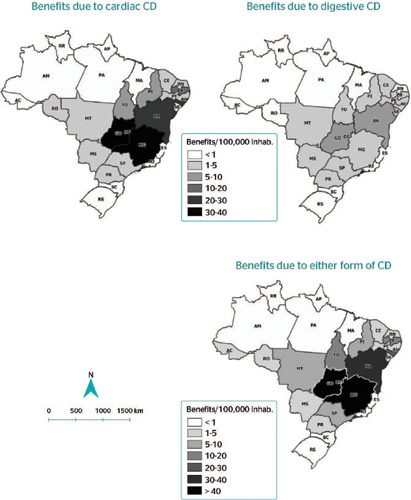
Distribution of benefits to people with Chagas disease (CD) per 100,000 inhabitants according to state and clinical form of the disease, Brazil, 2004-2016.

The Midwest macroregion had the highest relative frequency of benefits related to the cardiac (25.4/100,000 inhabitants), digestive (5.7/100,000 inhabitants), and general (31.1/100,000 inhabitants) forms of the disease (Figure 1). The Federal District had the highest relative frequency of benefits per 100,000 inhabitants for all 3 forms (cardiac: 39.7; digestive: 9.7; and general: 49.4), followed by the states of Goiás (cardiac: 39.1; digestive: 7.4; and general: 46.5) and Minas Gerais (cardiac: 35.6; digestive: 4.5; and general: 40.1) (Figure 2).

**Figure 3:**
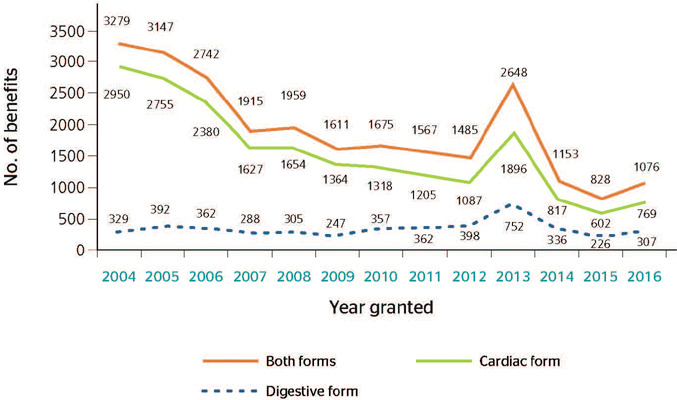
Distribution of benefits granted to people with chronic Chagas disease according to clinical form, Brazil, 2004-201

The number of benefits granted due to the cardiac and digestive forms of CD reduced over the study period, from 3279 (13.1%) in 2004 to 1076 (4.3%) in 2016 (Figure 3).

Male sex was 20% more associated with the cardiac form of CD (Table 1), and age > 29 years was also associated with a higher prevalence of the cardiac than the digestive form (Table 1). Residents of rural areas were 60% more likely to have the cardiac form (OR: 1.6; 95% CI 1.5-1.7). The Southeast and Midwest macroregions had the highest prevalence of benefits related to cardiac form, being 2.9 and 2.8 times higher than the South, which had the lowest prevalence (62.6%) (Table 1).

**Table 1:** Comparison of categorical variables among people who received social welfare benefits due to Chagas disease, according to clinical form, Brazil, 2004-2016

Variables	Cardiac form n (%)	Digestive form n (%)	Total n (%)	Crude OR (IC95%)	Adjusted OR (IC95%)
Sex (n = 25,085)					
Female	7431 (80.1)	1842 (19.9)	9273 (100.0)	1	
Male	12,993 (82.2)	2819 (17.8)	15,812 (100.0)	1.1(1.1-1.2)*	1.2 (1.1-1.2)*
Age range (n = 25,085) (years)					
≤ 29	391 (73.4)	142 (26.6)	533 (100.0)	1	
30-49	8549 (82.3)	1842 (17.7)	10,391 (100.0)	1.7 (1.4-2.1)*	1.8 (1.4-2.1)*
50-59	8757 (81.2)	2034 (18.8)	10,791 (100.0)	1.5 (1.3-1.9)*	1.6 (1.3-1.9)*
≥ 60	2727 (80.8)	643 (19.2)	3370 (100.0)	1.5 (1.2-1.9)*	1.7 (1.4-2.1)*
Residence zone (n = 25,085)					
Urban	12.766 (79.5)	3.285 (20.5)	16.051 (100.0)	1	
Rural	7.658 (84.8)	1.376 (15.2)	9.034 (100.0)	1.4 (1.3-1.5)*	1.6 (1.5-1.7)*
Geographic region (n = 25,085)					
South	393 (62.6)	235 (37.4)	628 (100.0)	1	
Southeast	9.468 (82.7)	1.976 (17.3)	11.444 (100.0)	2.9 (2.4-3.4)*	2.9 (2.4-3.4)*
North	238 (77.5)	69 (22.9)	307 (100.0)	2.1 (1.5-2.8)*	1.9 (1.4-2.6)*
Northeast	6.350 (81.1)	1.485 (18.9)	7.835 (100.0)	2.5 (2.1-3.0)	2.2 (1.8-2.6)*
Midwest	3.975 (81.6)	896 (18.4)	4.871 (100.0)	2.6 (2.2-3.2)*	2.8 (2.3-3.3)*
Type of benefit (n = 25,085)					
Assistance	1.352 (80.9)	320 (19.1)	1.672 (100.0)	1	
Pension	19.072 (81.5)	4.341 (18.5)	23.413 (100.0)	1.0 (0.8-1.1)	0.8 (0.7-1.0)

*p < 0.001.

OR = odds ratio

Although individuals with the cardiac form had a higher median age at disease onset, their age at work disability onset was lower than among those with the digestive form. In fact, the time elapsed between disease onset and work disability onset was longer among patients with the digestive form (Table 2).

**Table 2:** Comparison of numerical variables of social welfare beneficiaries according to clinical form of Chagas disease, Brazil, 2004-2016

Variables	Cardiac form	Digestive form	Kruskal-Wallis value	p-value
	Median (Q25%-Q75%)	Median (Q25%-Q75%)		
Age at illness onset (n = 25,085) (years)	45 (37-51)	44 (34-51)	55.9	<0.01
Age at disability onset (n = 23,151) (years)	50 (43-55)	51 (44-56)	5.7	0.01
Age at first benefits (n = 25,085) (years)	51 (44-57)	51 (44-57)	1.6	0.21
Time since work disability (n = 21,407) (days)	766 (177-2.219)	1.127 (227-3.653)	105.3	<0.01
Time as a contributor to social security (n = 23,365) (years)	6 (2-13)	7 (3-14)	77.8	<0.01

Q = quartile.

The median contribution time to social security was 7 years (25% quartile: 3 years; 75% quartile: 13 years). Beneficiaries with the digestive form contributed longer than those with the cardiac form (Table 2).

## DISCUSSION

In Brazil between 2004 and 2016, 25,085 individuals affected by CD received social security benefits, mainly men aged 50 to 59 years who resided in urban areas in the Midwest macroregion who were affected by the cardiac form of the disease, also called chronic Chagas cardiomyopathy. Male sex, rural residence, residence in the Southeast macroregion, and age between 30 and 49 years were associated with chronic Chagas cardiomyopathy. Individuals with chronic Chagas cardiomyopathy were older at disease onset and younger at work disability onset than those with the digestive form.

Men were the majority of the beneficiaries, and there was a 20% higher association between male sex and chronic Chagas cardiomyopathy, which was similar to the results of studies on CD mortality. Behavioral factors, lifestyle, and occupation might explain this finding, since rural-dwelling men are less likely to seek out health services, which reduces the chance of diagnosis and adequate treatment and increases the risk that chronic cases will become more serious forms that lead to work disability.^3,9^ Additionally, many of these workers perform or have performed manual labor, which requires greater cardiac activity.^18^

The low percentage of benefits among adults aged <29 years and the higher frequency of benefits among those aged 50 to 59 years reflect not only greater transmission control in recent decades, but the typically slow evolution of infection into chronic disease.^5^ Age > 29 years was more associated with chronic Chagas cardiomyopathy, with the greatest association being among those aged 30 to 49 years. Quantitative analysis revealed that those with the cardiac form had a higher median age at disease onset, but a lower age at work disability onset than those with the digestive form.

In fact, the time between disease onset and work disability onset was longer in digestive form cases. Another study linking chronic Chagas cardiomyopathy and work disability also found that individuals affected by this form become disabled at a younger age than those with other illnesses.^15^ In short, individuals with chronic Chagas cardiomyopathy present symptoms later, but it progresses to work disability more quickly than the digestive form.

The urbanization of CD has occurred throughout Latin America, mainly in the second half of the 20th century. In Brazil, it is estimated that 75% of those infected with CD live in urban areas.^19^ The social security data presented in this study clearly demonstrate this. With urbanization, the occupational profile of beneficiaries has also changed, ie, they are mainly involved in commercial activity. However, farming still occupies a prominent position among these social welfare beneficiaries.

It should be pointed out that, unlike the urbanization process of visceral leishmaniasis, for example, in which transmission has also urbanized, the vast majority of urban CD cases are chronic infections, acquired in the past by people who migrated from rural areas.^20,21^

More benefits due to chronic Chagas cardiomyopathy were granted to rural residents. Since engagement in primarily manual work, in addition to less access to health services, can accelerate the progression of cardiac cases to work disability, there is a greater need for benefits in this population. However, the digestive form of CD is not directly influenced by physical activity.^11,17^

The highest number of beneficiaries was in the Midwest macroregion, specifically, in the Federal District, followed by the states of Goiás and Minas Gerais. CD has been considered highly endemic in these places in the past and there is still a high prevalence of cases due to the chronicity of the disease.^3,5,20^ There are more cases of chronic Chagas cardiomyopathy in the Southeast and Midwest macroregions because they were more severely affected by CD in past decades. Considering the high transmission rates in these regions in the 1970s and 1980s and that approximately 20% to 30% of infected begin showing symptoms after 10 to 30 years, it can be inferred that these findings reflect the former epidemiological situation in Brazil.^5^

There was a higher prevalence of chronic Chagas cardiomyopathy among beneficiaries because this clinical form is the most disabling.^11,15,16,17^ In this study, chronic Chagas cardiomyopathy was responsible for the largest portion of both temporary and permanent disability benefits. It represents an important cause of work disability, especially among people whose occupations require intense physical effort or those entail personal or public risk, such as airline pilots, bus drivers, etc.^5,22^

The digestive forms of CD are generally less disabling, except in severe malnutrition or in situations requiring corrective surgical treatment.^11,22^ The undetermined or asymptomatic form involves no restrictions on work or activity. However, the patient’s clinical condition and work activity must be considered at the time of the medical examination.^11^

Social security enrollment is a legal link established between the system and contributors; contribution can be mandatory or optional. The “special case” status was the most prevalent type. This status is for small farmers and fishermen who work individually or with family members, but have no permanent employees.^23^ The number of unemployed beneficiaries was 20.2%. In a study on social welfare and AIDS during the same period, 51% of beneficiaries were unemployed, with the most prevalent age group being 20 to 39 years (49.8%).^24^

This comparison reflects two distinct epidemiological contexts. The vast majority (93%) of beneficiaries with CD participated in the labor market, having a more advanced age and having made a greater contribution to the social security system. Beneficiaries with AIDS were more socially vulnerable, including a large proportion of very young and unemployed individuals. This vulnerability is reflected in the benefit type: 26.5% of AIDS beneficiaries received social assistance, compared to only 6.7% of the CD patients in the present study.

The number of social security beneficiaries due to CD decreased during the study period. It should be pointed out that the impact of CD on Brazilian society as a whole has decreased since the 1980s and 1990s, when successful actions to combat the vector, associated with improved living conditions and migration to urban areas, caused the disease’s incidence to drastically decrease.^2,5^ Additionally, the disease’s high mortality, especially among older individuals, caused the prevalence to decline, which was reflected in a decreasing number of beneficiaries.^3^

This situation differs greatly from past decades, in which social security benefits for temporary or permanent disability due to CD were granted on a large scale. In 1979, for example, 0.3% of all social security benefits were granted to people with CD,^17^ although the impact of the disease was even greater in specific regions. In 1977, in the state of Goiás, 4.2 and 9.1% of all temporary and permanent disability benefits granted in urban areas, respectively, due to were due to chronic Chagas cardiomyopathy.^15^

It should be pointed out that data from the past had non-specific, variable, or descriptive coding, resulting in classifications such as “Functional heart disorders”, “Heart block”, or “Other forms of heart disease”, which underestimate the real prevalence and incidence of CD at that time.^15,17^

This study involves certain limitations that must be considered. Data consistency and completeness is an important limitation in any study that uses secondary data. However, because it involves the population’s social security and financial issues, the SUIBE information system has good data quality, consistency, and completeness.

Nevertheless, some points must be considered: (i) between 2004 and 2016, benefits were granted to 36,023 people, but, the clinical form of CD was not specified in 10,006 (27.8%), thus these data were not included in the study; (ii) benefits are granted based on a main cause, without including other comorbidities of interest. Tus, although cases of cardiodigestive CD were included, a distinction could not be drawn, since the cases are coded only by the main cause of disability. Moreover, a lack of previous research on the topic, which made comparisons impossible, could also be considered a limitation.

It is important to point out that there are workers with CD who are not enrolled in the social security system (eg, those who contribute to other retirement systems, such as the military and other public servants), as well as informal workers, individuals who do not work, and those who do not fulfill the basic requirements for assistance benefits. Such populations beyond the scope of the social welfare and social assistance system can lead to underestimation of the total number of people affected by the disease. Nevertheless, social security and assistance data can be useful as an indicator of morbidity, especially for neglected diseases such as chronic CD.

## CONCLUSIONS

The results showed that CD’s impact on Brazilian social welfare is mainly due to chronic Chagas cardiomyopathy. This form of the disease occurs mainly among men during their productive years who reside in rural areas in the Southeast macroregion of Brazil. Work disability occurs earlier in chronic Chagas cardiomyopathy than in the digestive form of the disease.
